# The Therapeutics of Creosote

**Published:** 1905-08-19

**Authors:** 


### THE THERAPEUTICS OF CREOSOTE.

Every new method of treatment which achieves any vogue at all, whether it be medicinal, dietetic, hydro-therapeutic or other, passes almost infallibly through a period of inflated importance followed by a reaction of disfavour quite as likely to be unjust as the earlier favourable exaggerations. But in course of time a more or less accurate mean of appreciation is reached and the remedy thenceforth assumes an assured position, be it high or low, in the recognised armament of medicine. The history of creosote is no exception to the rule, though it may be questioned whether its use has endured long enough for its value to have reached a stable equilibrium. None the less there is no doubt that creosote and its derivatives have a medicinal merit of no mean order.

In a review of the therapeutics of creosote Dr. J. M. French[1] points out that the best medicinal creosote is obtained by the double distillation of beechwood tar. Coal-tar creosote is a very different product, and much less suitable for internal administration than the other by reason of its irritant qualities. The great barrier to the use of the drug is its offensive taste and toxic character: the author advises that it should never be administered in aqueous solution, but either in milk, after being well stirred until it is emulsified, or in cod-liver oil or extract of malt. Creosote is eliminated chiefly by way of the lungs, and appears to act as a pulmonary antiseptic: its therapeutically active principle is guiacol, an oily liquid like creosote, but less irritant when taken internally. Its dosage is that of creosote, and its value in improving the appetite and digestion not inconsiderable.

The number of derivatives of creosote and guiacol upon the market is legion, but Dr. French directs particular attention to that known as thiocol. Thiocol is the guiacol-sulphonate of potassium, and, while possessing all, or nearly all, the merits of guiacol, has none of the disadvantages of the latter. Thus it is freely soluble in water and stable in the air, and can be exhibited in any form. Moreover, it is non-toxic, readily absorbed by the animal system, odourless and nearly tasteless, and absolutely unirritating to the stomach.

For some time following its introduction the use of creosote was confined almost entirely to the treatment of tuberculosis, especially when the site of the lesion was the lung. Whatever the precise *modus operandi*, there is little difference of opinion as to the value of its influence upon the outstanding symptoms of the disease. It is pretty generally admitted that creosote tends to allay the cough of phthisis and lessen the expectoration, fever, and night-sweats, while nutrition is aided by it and faulty gastric digestion to a considerable extent mitigated by the antiseptic properties of the drug. These general effects are common to all the various derivatives, selection being guided by the ease with which they are respectively tolerated. Dr. Rachford strongly recommends the external use of guiacol in the treatment of tuberculosis in infancy and childhood. For this purpose he employs an ointment consisting of guiacol one drachm, wool-fat two drachms, and lard three drachms, one drachm of the mixture being rubbed into the chest at night. Many French and American physicians speak very highly of creosote medication in the treatment of acute pneumonia. One of the foremost champions of the proceeding is Dr. I. L. Van Zandt, who has stated as his opinion that a large percentage of pneumonias are aborted by the remedy, almost all mitigated, and only a small residue unaffected.

Dr. Godson, writing in the *Birmingham Medical Review*, has reported his own experience of the value of creosote inhalations in cases of whooping-cough. He states that many cases are thus cured in a week, while the presence of creosote vapour in infected houses appears to exercise a most valuable prophylactic influence in favour of the unaffected members of the family. The vapour may be generated either by means of a steam-kettle or by evaporation over a lamp, but the constant presence of the vapour is essential to success. Without claiming that creosote is a specific for typhoid fever, many physicians hold that the type of the disease is rendered less severe by the exhibition of this drug, presumably by means of its capacity to diminish the abnormal putrefactive processes within the intestine. Again, Von Holstin asserts that pure creosote, administered in doses of from one to eight minims in milk thrice daily will overcome chronic constipation, and serve at the same time as a general tonic. Lastly, many observers speak well of guiacol as an external application for parotitis, orchitis, and epididymitis. It is applied in the form of an ointment containing 40 minims of guiacol to the half ounce of vaseline and the half ounce of wool-fat, the ointment to be rubbed in twice daily, and covered with protective to prevent evaporation.

[1] Merck's Archives, July 1905.

